# Carotid-body modulation through meditation in stage-I hypertensive subjects: Study protocol of a randomized and controlled study

**DOI:** 10.1097/MD.0000000000032295

**Published:** 2023-01-06

**Authors:** Tiago Rodrigues de Lemos Augusto, Juliana Peroni, Wandriane de Vargas, Priscilla Caroll Santos, Wendel Dantas, Roberta Lazari Padavini, Rodrigo Koch, Erlandson Saraiva, Marco Aurélio Vinhosa Bastos, Paulo de Tarso Müller

**Affiliations:** a Laboratory of Respiratory Pathophysiology (LAFIR), Maria Aparecida Pedrossian Universitary Hospital (HUMAP), Campo Grande, Mato Grosso do Sul, Brazil; b Mathematical Institute, Biostatistical department, UFMS, Brazil.

**Keywords:** carotid body, hypertension, meditation, variability, ventilation

## Abstract

Adjunctive therapy for hypertension is in high demand for clinical research. Therefore, several meta-analyses have provided sufficient evidence for meditation as an adjunct therapy, without being anchored on reliable physiological grounds. Meditation modulates the autonomic nervous system. Herein, we propose a hierarchical-dependent effect for the carotid body (CB) in attenuating blood pressure (BP) and ventilatory variability (VV) fine-tuning due to known nerve connections between the CB, prefrontal brain, hypothalamus, and solitary tract nucleus. The aim of this exploratory study was to investigate the role of CB in the possible decrease in BP and changes in VV that could occur in response to meditation. This was a prospective, single-center, parallel-group, randomized, controlled clinical trial with concealed allocation. Eligible adult subjects of both sexes with stage 1 hypertension will be randomized into 1 of 2 groups: transcendental meditation or a control group. Subjects will be invited to 3 visits after randomization and 2 additional visits after completing 8 weeks of meditation or waiting-list control. Thus, subjects will undergo BP measurements in normoxia and hyperoxia, VV measurements using the Poincaré method at rest and during exercise, and CB activity measurement in the laboratory. The primary outcome of this study was the detection of changes in BP and CB activity after 8 weeks. Our secondary outcome was the detection of changes in the VV at rest and during exercise. We predict that interactions between hyperoxic deactivation of CB and meditation; Will reduce BP beyond stand-alone intervention or alternatively; Meditation will significantly attenuate the effects of hyperoxia as a stand-alone intervention. In addition, VV can be changed, partially mediated by a reduction in CB activity. Trial registration number: ReBEC registry (RBR-55n74zm). Stage: pre-results.

## 1. Introduction

### 1.1. Background and rationale

Systemic arterial hypertension (HT) was the leading cause of cardiovascular mortality in 2010.^[1]^ Although the latest american college of cardiology & american heart association (2018) guidelines on HT do not recommend meditation,^[1]^ this opinion is not shared by the American Heart Association^[2,3]^ and the International Society of Hypertension.^[4]^ However, the mechanisms underlying these effects are largely unknown.

Meditation can modulate the autonomic nervous system (ANS),^[5–8]^ although a recent meta-analysis did not support this premisse.^[9]^ Herein, we propose a role for the carotid body (CB), the principal oxygen chemoreceptor in humans, playing crucial functions in maintaining respiratory, cardiovascular, and neurohumoral homeostasis.^[10]^ The rationale for this hypothesis is supported by the meaningful nerve connections between the CB, hypothalamic areas, and *nucleus tractus solitarii*,^[11–14]^ with a prevailing effect on increasing sympathetic activity in normoxic conditions due to abnormal hypertonicity in HT.^[12,14]^ In fact, there is some evidence of a link between meditation and balance of neurotransmitters in the hypothalamus.^[15–17]^ We speculate that the connection between meditation, the hypothalamus-pituitary-adrenal axis (HPA), and CB activity would involve stabilization of the HPA axis, as has been proposed with respect to yoga^[18–20]^ or transcedental meditation^[21]^–and a highly coordinated engagement of brain structures such as the prefrontal cortex, amygdala, and hippocampus (hyperachical-dependent activity). Of note, the prefrontal cortex, amygdala, and hippocampus gray matter have been shown to change with yoga/meditation.^[22–25]^ Interestingly, hypertonicity of the CB^[12,26–29]^ and increased size and weight of the CB^[30–35]^ are common findings in HT. However, inconsistent evidence has revealed a decrease in systemic blood pressure (BP) through CB deactivation in response to hyperoxia in hypertensive subjects,^[36–38]^ suggesting that hyperactivity of the CB may be mechanistically associated with sympathetic dysregulation.^[12,39]^ In addition, CB and BP are closely linked to ventilatory variability,^[40–42]^ and lifestyle interventions can reduce ventilatory variability during exercise.^[43]^ Acute changes in breathing variability have also been demonstrated during meditation.^[44]^

### 1.2. Study aims and outcomes

Thus, suspected hyperactivity of CB beyond hypoxic states would be a straightforward pathway to investigate mechanisms underpinning anti-stress therapies in HT, which could theoretically decrease the hierarchical-dependent influence of CB. This exploratory study aimed to investigate the putative role of CB in the decrease in BP and changes in ventilatory variability that could occur in response to meditation. Hence, the primary outcome of this study was to detect changes in BP and CB activity after 8 weeks of meditation. The secondary outcomes were changes in rest and exercise ventilatory variability. We anticipate that interactions between hyperoxic deactivation of CB and meditation at 8 weeks; Will reduce BP beyond stand-alone intervention, or alternatively; Meditation will significantly attenuate the effects of hyperoxia as a stand-alone intervention for BP. In addition, breathing variability could change, partially mediated by a reduction in CB activity.

## 2. Material and Methods

### 2.1. Study design and setting

**Figure 1.**
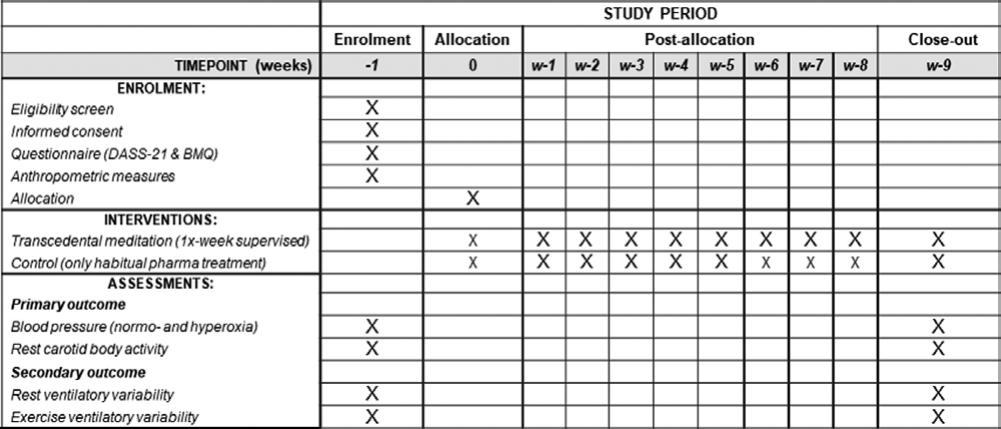
Schedule of enrollment, interventions, and assessments from SPIRIT Guidelines. DASS-21 = Depression, anxiety and Stress Scale, BMQ = Brief medication questionnaire.

This was a prospective single-center, parallel-group, randomized, controlled trial with concealed allocation and with 8 weeks of follow-up analyzed using an intention-to-treat approach. The study will be performed at the Laboratory of Respiratory Pathophysiology (LAFIR) at the Respiratory Division of the University Hospital (HUMAP) and the comprehensive care and monitoring of persons with hypertension in primary health care. This will be performed according to the SPIRIT (2013) guideline checklist and recommendations. A Schedule of enrollment, interventions, and assessments from the SPIRIT guidelines is shown in Figure 1.

### 2.2. Participants’ timeline

This study began in October 2022 and is expected to end after 18 months. The subjects will be invited to 3 visits after randomization and 2 additional visits after completing 8 weeks of a meditation trial. Anthropometric, demographic, and self-report data will be collected on the 1^st^ visit, and familiarization with the laboratory procedures will be performed. In addition, a preparatory briefing will be delivered by a trained researcher who is not involved in the laboratory work. The preparatory briefing consisted of an hour-long talk about HT diagnosis and management. The 2^nd^ visit to the lab will be dedicated to studying resting ventilatory variability (RVV), BP measurements under normoxic or hyperoxic trials conditions (NHT), and resting carotid-body activity (RCBA). The 3^rd^ visit will involve symptom-limited maximal cardiopulmonary exercise testing (CPET) and exercise ventilation variability (EVV) acquisition. The same sequence for the in-lab physiological study will be performed after 8 weeks of meditation or control participation (Fig. 1).

### 2.3. Ethical concerns and trial registration

This study was registered and approved by the Human Research Ethics Committee of our institution (CAAE number: 53122821.6.0000.0021). Individuals will be recruited through social media and banners and should agree to participate and sign an informed consent form. The study followed best practices in research and medical conduct and the recommendations of the Declaration of Helsinki. Rebec registration: Rebec RBR-55n74zm.

### 2.4. Sample details

#### 2.4.1. Diagnostic criteria for stage I hypertension.

Stage 1 HT was diagnosed based on both the last “Brazilian Guidelines of Hypertension”^[45]^ and the “2018 ESC/ESH Guidelines for the management of arterial hypertension.”^[46]^ Thus, a systolic blood pressure (SBP) between 140 to 159 mm Hg and/or diastolic blood pressure (DBP) between 90 to 99 mm Hg define stage 1 HT after carefully measuring technical recommendations.^[45]^

#### 2.4.2. Inclusion criteria.

Subjects will be enrolled in this trial if they meet the following inclusion criteria:
(1) both genders.
(2) age between 30 to 70 years.
(3) previous physician diagnosis of primary hypertension or taking at least 1 antihypertensive medication.
(4) minimum 1-year diagnosed stage 1 HT.
(5) intake of antihypertensive medication unchanged for at least 2-months (see the Brief Medication Questionnaire).
(6) not engaged in regular exercise programs (3 or more times per week) in the last 3 months before the study; and.
(7) being able to read, speak, and understand Portuguese.

#### 2.4.3. Exclusion criteria.

Subjects will be excluded according to the following criteria:
(1) subjects who did not adhere to the experimental protocol.
(2) had an important comorbidity, such as heart failure ≥ NYHA I, COPD, cognitive impairment (mini-mental exam < 27 points) or psychiatric disease, previous stroke, neuromuscular disease, asthma, uncontrolled diabetes, peripheral vascular disease, obesity grade III, renal failure of stage > 2 with glomerular filtration rate < 60 mL/minutes/1.73 m2, sleep apnea, malnutrition (BMI < 18.4) or other diseases that interfere with the performance of the protocol.
(3) missing more than 2 weekly based supervised meditation sessions.
(4) secondary hypertension.
(5) contraindications to perform a symptom-limited maximal exercise test; and.
(6) regular meditation practice.

#### 2.4.4. Randomization and assignment.

**Figure 2.**
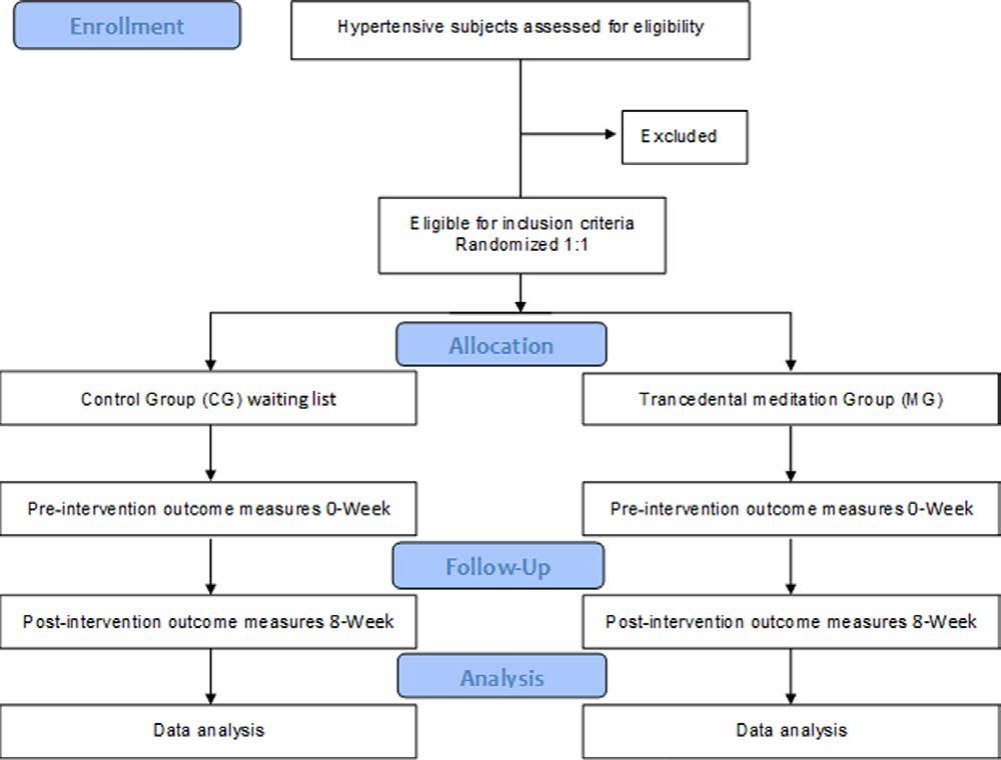
CONSORT 2010 flow diagram for the study.

Recruitment, randomization, and assignment will be conducted by 4 researchers. Two researchers will be involved in enrolling subjects in the comprehensive care and monitoring of persons with hypertension in primary health care. Another researcher randomized the subjects into 2 parallel groups through block randomization (block size:8) using computer-generated random numbers (random.org). A 4^th^ researcher assigned the participants to 2 groups at an allocation ratio of 1:1. The opaque envelopes enclosing the study group allocation will be numbered consecutively and sealed. Once a patient signs the informed consent form and baseline data acquisition has been concluded, the study researcher will open the envelope with the lowest remaining serial number, and the patient will accordingly be allocated to a group. The subjects will be randomly assigned to 2 groups: a transcendental meditation group and a control group (CG). The CG will remain under pharmacological treatment for 8 weeks and will be set up on a waiting list. Both groups were contacted weekly for adherence and adverse events. The CONSORT flowchart of the participants is shown in Figure 2. A careful explanation of the study’s potential social and academic pertinence and benefits will be provided during the consent action to encourage participant adherence to the intervention and assessments.

#### 2.4.5. Blinding.

The 4 researchers involved in recruitment, randomization, and assignment were blinded to the data collection in the lab. In turn, the additional 3 researchers assigned to the lab will be blinded to the randomization and assignment. The subjects and researchers will be strictly advised not to chat or provide clues as to which group they belong. The subjects at the lab will be blinded to the normoxic or hyperoxic gas trials, which will be delivered with similar gas cilinders. The statistician will also be blinded to all the protocols and data collection. Finally, participants were blinded to the study hypotheses.

#### 2.4.6. Sample size.

Since there are no previous studies on CB activity after meditation, our a priori sample size calculation was based on a previously cited pilot study on RCBA ^[47]^. Considering an effect size of 0.25, a minimum of 30 subjects would be necessary to detect a significant change in chemoreception based on within-group analysis. In addition, the sample size was calculated based on a pilot study. The pilot study comprised 12 normotensive/prehypertensive healthy subjects and showed a standard deviation of the mean difference for SBP ± 4.2 mm Hg, during 2 different trial days (see Supplemental Digital Content, http://links.lww.com/MD/I272), with the oscillometric method and under the same experimental conditions (normoxic, see subsection 2.5.7). Based on a repeated measure ANOVA design with 1 between-group factor (Meditators vs Control) and 2 within-groups factors (Gas type and Time), with 4 averaged BP measures [pre-normoxic (T1), pre-hyperoxic (T2), post-normoxic (T3), post-hyperoxic (T4)] for each subject, upon resting condition, 30 subjects per group would be adequate in a 2-sided study to set an error type I = 0.05 and *β* = 0.80. Considering a 20% dropout and exclusion rate, a minimum total sample size of 36 per group was required. This sample size is concordant with several studies on the effects of meditation on hypertension.^[47]^

### 2.5. Procedures and interventions

#### 2.5.1. Health status and antropometric data.

The participants will be interviewed for health status, smoking status, previous medical examinations, and demographic data. Anthropometric data were recorded using a previously calibrated Welmy scale (Model: 110 CH G, Welmy, Brazil). The body mass index (BMI) was calculated.

#### 2.5.2. The DASS-21: depression, anxiety, and stress scale.

We used the public domain DASS-21 questionnaire to characterize perceived depression, anxiety, and stress. This instrument has 21 questions with a 0 to 4 Likert scale, is validated for the Portuguese language, and shows high internal consistency (Cronbach’s alpha was.92 for depression,.90 for stress, and.86 for anxiety).^[48]^

#### 2.5.3. The brief medication questionnaire (BMQ): adherence.

This questionnaire was intended to assess adherence to pharmacological treatment with antihypertensive medications. This instrument has been validated for the Brazilian Portuguese language and is in the public domain, with 80% sensitivity and 100% specificity in the regimen domain.^[49]^ The BMQ in its validated Brazilian version was superior to the Morisky-Green questionnaire.^[49]^

#### 2.5.4. Preparatory briefing about HT and meditation.

On the 1^st^ visit, the subjects allocated to both groups will be invited to an introductory explanation of hypertension after laboratory instrument familiarization. A researcher not directly involved in lab data collection will introduce fundamental knowledge about essential hypertension, the autonomous nervous system, and treatment. The CG will only receive information about hypertension and the meditation group will receive an additional preparatory briefing about meditation during the 1^st^ meditation session.

#### 2.5.5. Familiarization.

During the 1^st^ visit, all randomized subjects will be invited to the laboratory to perform several normoxic BP measurements using naso-oral masks and accessories under paced breathing. Submaximal testing (up to Borg 5) with a cycle ergometer will also be performed with a naso-oral mask intended to desensitize discomfort.

#### 2.5.6. RVV.

**Figure 3.**
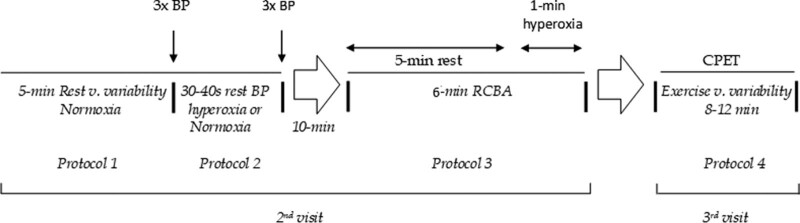
Data collection timeline at the lab scheduled before allocation and repeated upon intervention completion. BP= blood pressure, v = variability, RCBA = resting carotid body activity, CPET = cardiopulmonary exercise testing.

During the 2^nd^ visit, the subjects will be admitted to a climatized lab room at ~7:00 hours after a light breakfast without stimulant foods and will be instructed to remain calm during the study protocol. They will be monitored using a QUARK metabolic system (QUARK CPET, COSMED, Rome, Italy, 2016) for breath-by-breath oxygen consumption (*V*´O_2_), minute ventilation (*V*´_E_), tidal volume (*V*_T_), breathing frequency (*f*R), end-tidal carbon dioxide (*P*_ET_CO2), and inspiratory/expiratory time (s) using a low-resistance calibrated turbine (COSMED, Rome, Italy, 2016). The analyzer was calibrated with 2-point precision gases (GAMA GASES, São Paulo, Brasil). The turbine was attached to a non-rebreathing 2-way valve connected to a naso-oral mask attached to the head with head straps (Hans-Rudolph Inc., USA, 2036 series, 2019). All trials will be registered in real time and saved for posterior analysis in accordance with previously published methods.^[50]^ Resting variability was measured using our previously described method.^[51]^ First, a non-linear evaluation of breath-by-breath signals using an *in house* Poincaré analysis algorithm will be performed using the “R^®^” free software program (http://www.R-project.org/) to calculate SD1 and SD2 for minute ventilation, tidal volume, and breathing frequency. The resting trial was performed in a seated position for 5 min (Protocol 1, Fig. 3).

#### 2.5.7. BP measurements.

Blood pressure will be measured based on replicated tests under the same described metabolic and ventilatory RVV monitoring. The arm was positioned at the heart level, and the NHT sequence was preceded by baseline BP measures (3x, 1-minute apart) immediately after the 5-minutes RVV acquisition (Protocol 1, Fig. 3). The target is a minimum of 3 BP measures differing within < 5 mm Hg and no BP measure differing within > 10 mm Hg^[1,52]^ between 3, 1-min apart, successive oscillometric measurements (Multiparametric Monitor, Dixtal-Philips^®^, Manaus, Brazil, 2015). The measurements will be preceded by a 3 to 4 minutes recovery only for hyperoxic trials, ensuring that the BP and *P*_ET_O_2_ return to the baseline value. Aditional measurements will be performed only if the first 2 readings differ by > 10 mm Hg, and BP will be recorded as the average of the last 2 readings.^[1]^ The noninvasive blood pressure (NIBP) system was properly calibrated at regular 3-month intervals using an NIBP simulator (Comprehense NIBP simulator^®^, Brazil, 2018). The trials will be repeated during normoxic and subsequently hyperoxic gas delivery to avoid residual effects of CB deactivation under normoxic conditions. The lights will be dimmed to ensure stable measurements and no interaction with the participants will occur during the assessment.

#### 2.5.8. NHT.

The NHT will be performed under paced breathing frequency (20 breaths minute^-1^) with controlled inspiratory time (duty cycle ~ 0.3) throughout metronome pacing using incentive screens (Bounce Metronome, USA, 2015). Pacing breathing is important to avoid lower respiratory frequency and baroreceptor modulation with an acute reduction in blood pressure, as suggested in essential hypertension.^[53]^ The NHT will be performed through 21% or 100% FIO_2_ (in this order) through gas cylinders (White-Martins™, Campo Grande, MS, Brazil), delivered through a Douglas bag connected to a *Y*-shaped 2-way non-rebreathing valve (Hans-Rudolph Inc., USA, 2036 series, 2019) during 30 to 40 seconds trials, because previous studies have shown that the CB hyperoxic deactivation effect in BP is lost after this time, presumably due to systemic arterial intimal oxidative stress, which rebounds vasoconstriction^[27,36]^; Of note, the time delay for oscillometric BP measurement is reliably between 30 to 40 seconds for our chosen device. The participants will be permitted breaks from paced breathing between interventions (Protocol 2, Fig. 3).

#### 2.5.9. RCBA.

Resting CB activity will be measured through a hyperoxic trial (FIO_2_ = 100%) after ensuring stable minute ventilation (5-minutes of normoxic calm breathing). Baseline minute ventilation is the average value of the last 60 seconds of breath-by-breath stable ventilatory signal. During the next 1-minute hyperoxic trial delivered through a non-rebreathing system, the nadir of 10 seconds averaged minute ventilation was measured.^[54]^ The test was repeated 3 times (3-minutes apart), and the average value was considered (Protocol 3, Fig. 3). The lowest value is taken as an index of basal CB activity, and a greater reduction in minute ventilation corresponds to a greater tonic input from the carotid body.^[54]^

#### 2.5.10. CPET and EVV.

Incremental CPET followed the routine of our laboratory and has been previously published.^[55,56]^ First, after strict recommendations for abstinence from stimulants and depressants and using only habitual medication, the individuals will be stimulated to pedal to 60 cycles minute^-1^ after 2-minutes of rest and 2-minutes of warm-up toward maximum work rate tolerance, meaning that they cannot pedal for more than 10 seconds over 50 cycles minute^-1^ under the stimulus of the examiner, and present subjective signs of intense exhaustion. The initial power of “0” W during the warm-up was increased by 16 W/minute^-1^. Breath-by-breath oxygen consumption (*V*´O_2_), exhaled carbon dioxide (*V*´CO_2_), minute ventilation (*V*´_E_), respiratory rate (*f*R), and tidal volume (*V*_T_) components were measured using a QUARK CPET metabolic chart (COSMED, Italy, 2016), calibrated at 2 moments with high precision gases (GAMA-GASES, São Paulo, Brazil) before each test. Heart rate (HR) and rhythm will be monitored using an ECG system (COSMED® Tx12 ECG, Italy, 2016), integrated into the metabolic system, and programmed to control an electromagnetically braked cycle ergometer, the Imbrasport CG-400 model (Imbramed^®^, Porto Alegre, Brazil, 2017). Continuous peripheral digital oximetry monitoring (COSMED, Italy, 2018) was also performed. During CPET, BP is measured using the auscultatory technique based on the Korotkoff method. Measurements will be performed at baseline, at 2-minute intervals, and at the exercise peak. EVV will be measured as described for RVV, taking into account the exercise values for *V*´_E_, *V*_T_, *f*R, and *P*_ET_CO2 (Protocol 4, Fig. 3). As variability metrics depend on the number of respiratory cycles during the variable exercise period, we divided SD1 and SD2 by the number of breath-by-breath measured data collected for each subject (SD1/n and SD2/n) as recommended.^[51,57]^

#### 2.5.11. Transcedental meditation (TM).

Transcendental meditation follows previously explored methods.^[58,59]^ A certified trainer will teach the TM technique to the group in the standard 4-day course preceded by an introductory briefing. The subjects will be widely informed about the method, receiving a 1-hour group-based introductory briefing about the method in the 1st session, followed by a personal interview, personal instruction session, and group verification sessions on 3 consecutive days following personal instruction. To verify the correctness of TM practice, group-based TM sessions will be performed once a week, guided by a senior professional therapist in a 20-minutes session in an appropriate and comfortable room while sitting comfortably with their eyes closed. During the week, the subjects will be trained to perform 2 20-minutes unsupervised sessions at home each day. TM will be implemented in a more cognitive fashion, with passive mental focusing associated with slow breathing. Predefined weekly contact by phone will be performed to check for adherence and adverse effects.

### 2.6. Outcomes

For participants who dropped out of the study at any time after randomization, researchers will use contact details to encourage them to undergo the end-study outcome assessments (8-weeks). Thus, outcomes will be assessed before and after the intervention period, regardless of attending or completion status.

#### 2.6.1. Primary outcomes.

The primary outcomes were as follows:
(i) BP changes under normoxia.
(ii) BP changes under hyperoxia, and.
(iii) changes in RCBA after 8 weeks of TM compared with control subjects. Any BP difference for normoxic or hyperoxic trials > ± 5.9 mm Hg, as determined by our reliability study, after 8 weeks will be considered a clinically significant result (Figure S2 and S3, Supplemental Digital Content, http://links.lww.com/MD/I272). To the best of our knowledge, there has been no reproducibility study of the RCBA in adults with hypertension.

#### 2.6.2. Secondary outcomes.

The secondary outcomes were
(i) changes in RVV and.
(ii) changes in EVV, both after 8 weeks of meditation, c006Fmpared to control subjects. Any SD1 difference > 1.2 L (Figure S1, Supplemental Digital Content, http://links.lww.com/MD/I272), after 8 weeks of meditation will be considered a clinically significant result.

### 2.7. Data management

Data were downloaded on standardized forms identified by subject number and trial ID. All the data were managed using the RedCap platform. A researcher will conduct an audition of missing or inaccurate data. Data will be backed up daily by automated export procedures from secure servers of the Hospital Maria Aparecida Pedrossian (HUMAP).

### 2.8. Scheme for study retention

Participants will be contacted weekly via phone calls or text messages intended to promote adherence to the study and reschedule missed sessions as much as possible. Attendance will be monitored through session frequency, and adherence will be accounted for as a percentage of fully accomplished interventions.

### 2.9. Safety assessments

All lab tests, evaluations, and interventions in the present study will be performed in a safe environment with a fully equipped emergency resuscitation trolley. Any adverse events that occurred during the study will be monitored to ensure patient safety, and all participants will be monitored. All adverse events will be collected and included in the research reports, which will be forwarded to the Ethics Committee of our institution.

### 2.10. Statistical analysis plan

The 2 groups will initially be compared at baseline in terms of their characteristics, using the chi-squared test for categorical variables and *t*-tests for continuous measures, after Shapiro–Wilk tests for normality. Descriptive analysis will be performed using frequencies and percentages to obtain clinical, anthropometric, and sociodemographic conditions. Two-way ANOVA for repeated measurements will be applied considering between-factors (2 groups) as independent variables and 2 within-factors for BP measurements (gas type and time) as dependent variables (pre-normoxic, pre-hyperoxic, post-normoxic, and post-hyperoxic). A mixed-design with repeated measures will be applied for RCBA, RVV, and EVV as dependent variables in comparing week “0” with week “8.” A post hoc analysis using the Bonferroni procedure, taking into account standard sphericity, will be performed. If this criterion is violated through the Mauchly test, we use the Greenhouse-Geisser correction. In order to explore possible change effects after meditation, coefficients of correlation through Pearson product-moment, including ∆ BP, ∆RVV and ∆ EVV as dependent variables and, ∆RCBA as independent variable, will be performed as percentage of change (∆= (“8 week–“0 week/0 week”)_ *_ 100. Effect sizes (Cohen-d) will also be presented to show the magnitude of our findings, using the scores of 0.20 as small, 0.50 as a medium, and 0.80 as large. Statistical Package for Social Sciences software (version 17.0; IBM Corp, Armonk, NY) was used for all statistical analyses. The study will adopt a *P-value* ≤ 0.05 as statistically significant.

## 3. Discussion

This is the first study to explore the possible interaction between carotid body activity and blood pressure after 8 weeks of TM. In addition, we will study ventilatory variability as a secondary endpoint to verify the possible effects of TM on ventilation.

Some physiological pathways have been reported to undergo modulation and change by meditation in the context of hypertension, such as improvement in ANS imbalance,^[5–7,60]^ HPA axis modulation,^[18]^ increased cardiorespiratory coherence,^[16]^ improvement in vascular function,^[61]^ and increased gene transcription, including antioxidative up-regulation.^[62]^ Concurrently, some of these pathways are associated with the pathophysiology of neurogenic hypertension, presumably triggered by persistently disturbed cortical impulses (neocortex and cingulate gyrus) through chronic stress and mental exhaustion.^[63]^ Based on recent scientific studies,^[12,14]^ we hypothesized that the hierarchical-dependent sympathetic dysfunction of CB in chronic hypertension could be modulated by an anti-stress intervention, such as meditation, presumably via interaction with higher cerebral levels. There is plenty of evidence on CB dysfunction in HT, and some studies advocate an upregulation of sympathetic effects on blood pressure and ventilatory control, beyond hypoxic-hypercapnic stimulus^.[31,36,64]^ Evidence of modulation of CB by meditation is an important step in establishing connections between anti-stress treatment and CB dysfunction, which could explain, for instance, the inter-individual variability of responses to hypoxia/hypercapnia in the presence of chronic hyperventilation syndrome.^[65,66]^

## 4. Strengths and limitations of the study

In this study, we followed strict pacing of the respiratory pattern during the NHT tests, with ventilometry and end-tidal carbon dioxide monitoring. Our aim was to sustain a similar ventilatory pattern before and after meditation during the BP measurements. This is highly recommended because respiratory pattern changes lead to ANS modulation, especially in hypertensive subjects, with hemodynamic and cardiac rhythm consequences.^[67]^ In addition, we designed this study with special concerns regarding antihypertensive medication adherence (BMQ) and repetitive BP measures (to minimize the chance of white coat effect).^[52]^ Training with paced breathing should also be performed to reduce psychological stress. Despite careful repetitive BP measurements and a previous reliability study, the main limitation of this study is the absence of beat-by-beat noninvasive BP monitoring.

## Acknowledgments

The NAR group was on a Self-Realization Fellowship (Professor Adão Nazareno Marques Barros). Maria Aparecida Pedrossian Hospital (HUMAP, Campo Grande, Brazil). The HUMAP Superintendent is Professor Cláudio Cézar da Silva.

## Author contributions

**Conceptualization:** Tiago Rodrigues de Lemos Augusto, Rodrigo Koch,Juliana Peroni, Wandriane de Vargas, Roberta Lazari Padavini, Erlandson Saraiva, Marco Aurélio Bastos, Paulo de Tarso Müller.

**Formal analysis:** Paulo de Tarso Müller, Erlandson Saraiva.

**Funding acquisition:** Paulo de Tarso Müller.

**Investigation:** Tiago Rodrigues de Lemos Augusto, Rodrigo Koch, Juliana Peroni, Wandriane de Vargas, Roberta Lazari Padavini, Priscilla Carol Santos, Weldel Dantas, Erlandson Saraiva, Marco Aurélio Bastos, Paulo de Tarso Müller.

**Methodology:** Tiago Rodrigues de Lemos Augusto, Erlandson Saraiva, Marco Aurélio Bastos, Paulo de Tarso Müller.

**Project administration:** Roberta Lazari Padavini, Paulo de Tarso Müller.

**Resources:** Paulo de Tarso Müller.

**Supervision:** Roberta Lazari Padavini, Paulo de Tarso Müller.

**Writing — original draft:** Tiago Rodrigues de Lemos Augusto, Paulo de Tarso Müller.

**Writing — review & editing:** Tiago Rodrigues de Lemos Augusto, Rodrigo Koch, Juliana Peroni, Wandriane de Vargas, Roberta Lazari Padavini, Erlandson Saraiva, Proscilla Caroll Santos, Wendel Dantas, Marco Aurélio Bastos, and Paulo de Tarso Müller.

## Supplementary Material


