# Supplementary material for: Carotid-body modulation through meditation in stage-I hypertensive subjects: Study protocol of a randomized and controlled study
Source: Medicine (Baltimore). 2023 Jan 6;102(1):e32295. doi: 10.1097/MD.0000000000032295 (PMC9829266; doi:10.1097/MD.0000000000032295)
Supplement: Supplementary file 1 [file medi-102-e32295-s001.pdf]

STUDY PROTOCOL

SUPPLEMENTAL DIGITAL CONTENT

Carotid-Body modulation through meditation in stage-I hypertensive subjects: study protocol of a randomized and controlled study

Authorship order: Tiago Rodrigues de Lemos Augusto, Juliana Peroni, Wandriane de Vargas, Priscilla Caroll Santos, Wendel Dantas, Roberta Lazari Padavini, Rodrigo Koch, Erlandson Saraiva, Marcos Aurélio Bastos, Paulo de Tarso Müller.

SUPPLEMENTAL DIGITAL CONTENT

1.Reproducibility for Rest Ventilatory Variability (RVV).

Twelve normotensive/prehypertensive subjects underwent an RVV reproducibility study second the following protocol, performed twice, one-week apart, under the same conditions of the laboratory. The subjects were admitted at a climatized lab room at ~7:00 hs after a light breakfast without stimulant foods, and were instructed to remain calm during the study protocol. They were monitored through a QUARK metabolic system (QUARK CPET, COSMED, Rome, Italy, 2016) for breath-by-breath oxygen consumption ($V'O_2$), minute-ventilation (V'_E), tidal volume (V_T), breathing frequency (*f*R), end-tidal carbon dioxide ($P_{ET}CO2$) and inspiratory/expiratory time (s), using a low-resistance calibrated turbine (COSMED, Rome, Italy, 2016). The analyser was calibrated with 2-point precision gases (GAMA GASES, São Paulo, Brasil). The turbine was attached to a non-rebreathing two-way valve connected to a naso-oral mask attached to the head with head straps (Hans-Rudolph inc., USA, 2036 series, 2019). All the trials were registered in real-time and saved for posterior analysis, in accordance with previous published methods [S1]. Resting variability will be measured based on our previously described method [S2]. First, non-linear evaluation of breath-by-breath signal using an *in house* Poincaré analysis algorithm will be performed using the "R®" free software program (http://www.R-project.org/) to calculate the SD1 for minute-ventilation. The resting trial was performed at a seated position for 5 min.

As results, SD1 for minute-ventilation (L/min) showed an average difference between Test 1 and Test 2 of 0.08±0.9 L, and limits of agreement (LOA) of -1.8/1.7 L (See Supplementary Figure 1).

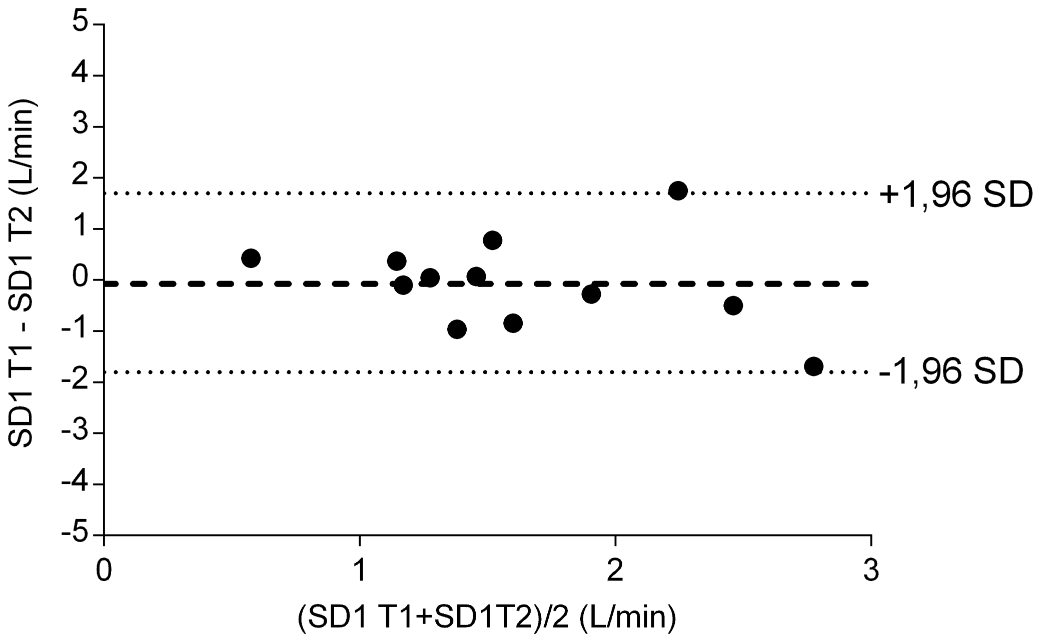

S. Figure 1 Bland-Altman plot for Poincaré variability analysis in a test-retest design for minute-ventilation SD1. T1 = data collection at Time 1 and T2=data collection at Time 2.

2.Reproducibility for blood pressure without and with paced breathing.

Blood pressure were measured based under the same described metabolic and ventilatory RVV monitoring. The arm was positioned at the heart level and BP measures (3x, 1-min apart) only under normoxic conditions were measured, immediately after the 5-min RVV acquisition. The target was a minimum of three BP measures differing within < 5 mmHg and none BP measure differing within > 10 mmHg [S3, S4] between three, 1-min apart, successive oscillometric measurements, (Multiparametric Monitor, Dixtal-Philips®, Manaus, Brazil, 2015). Aditional measurements were performed only if the first two reading differ by > 10 mmHg and BP was recorded as the average of the last two BP readings [S3]. After 10-min resting, the subjects underwent the same protocol for blood pressure measurements under paced breathing frequency (20 breaths min^{-1}), with controlled inspiratory time (duty cycle~0.3) throughout metronome pacing with incentive screens (Bounce

Metronome, USA, 2015). The lights were dimmed to ensure stable measurements, and no interaction with the participants will occur during the assessment.

As results, blood pressure without paced breathing showed an average difference between Test 1 and Test 2 of -0.58±4.5 mmHg, and limits of agreement (LOA) of -9.5 /8.5 mmHg (See Supplementary Figure 2). For blood pressure under paced breathing, the average difference between Test 1 and Test 2 was -0.50±4.2 mmHg, and limits of agreement (LOA) of -8.6/7.6 mmHg (See Supplementary Figure 3). The blood pressure during paced breathing was measured under similar average minute-ventilation in comparing Test 1 (14.8±5.4 L) and Test 2 (15.0±6.3 L) trials (p-value=0.867).

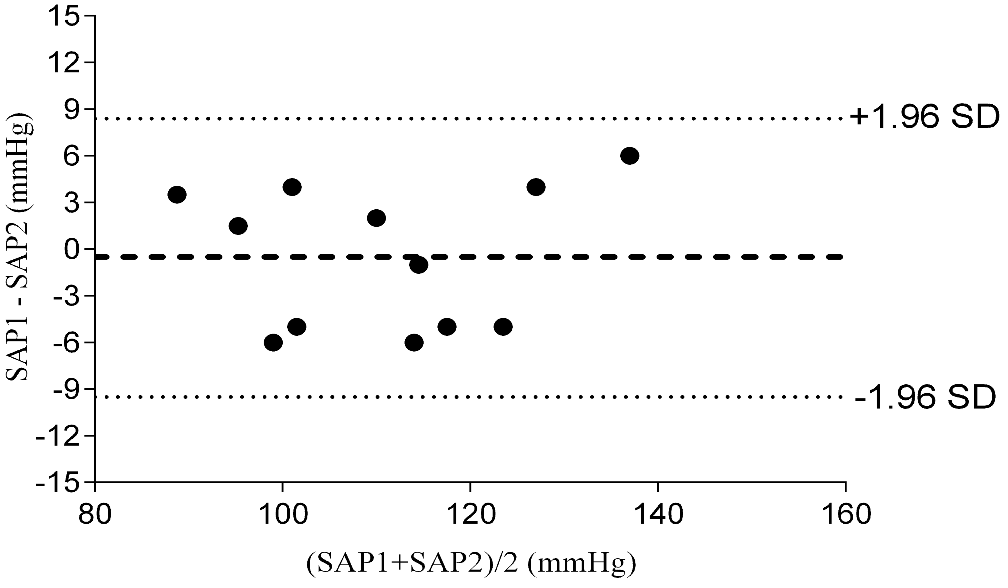

S. Figure 2 Bland-Altman plot for reproducibility analysis <u>without</u> paced breathing, in a test-retest design for systolic blood pressure (SBP). Data collection at Time 1 and Time 2.

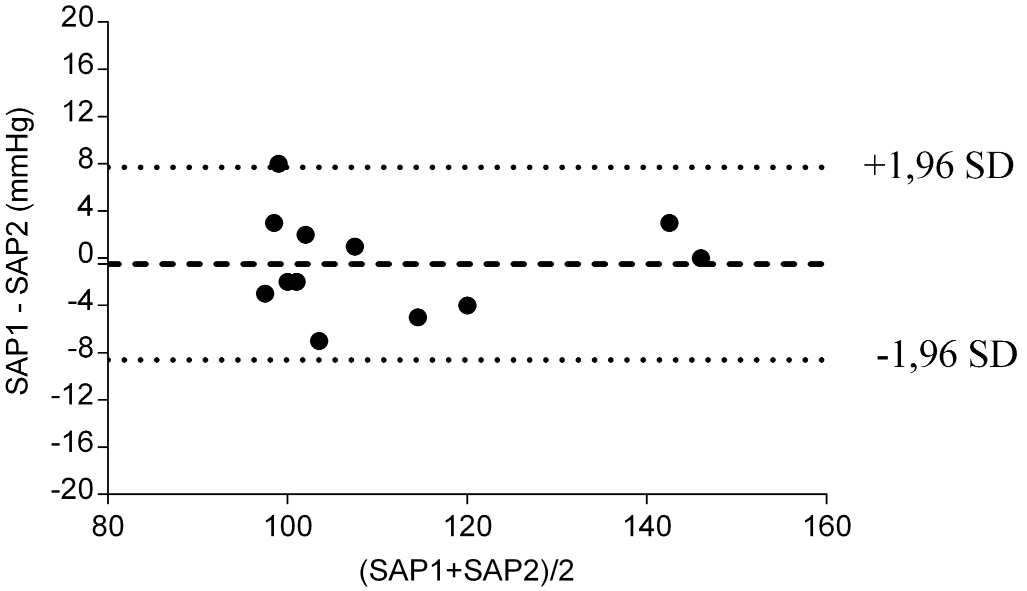

S. Figure 3 Bland-Altman plot for reproducibility analysis with paced breathing, in a test-retest design for systolic blood pressure (SBP). Data collection at Time 1 and Time 2.

3. Supplementary References

[S1] Jiang Y, Costello JT, Williams TB, et al. A network physiology approach to oxygen saturation variability during normobaric hypoxia. Exp Physiol. 2021; 106: 151-159. doi: 10.1113/EP088755.

[S2} Muller PT, Utida KAM, Augusto TRL, et al. Left ventricular diastolic dysfunction and exertional ventilatory inefficiency in COPD. Respir Med. 2018; 145: 101-109. doi: 10.1016/j.rmed.2018.10.014.

[S3] Whelton PK, Carey RM, Aronow WS, et al. ACC/AHA/AAPA/ABC/ACPM/AGS/APhA/ASH/ASPC/NMA/PCNA Guideline for the Prevention, Detection, Evaluation, and Management of High Blood Pressure in Adults: Executive Summary: A Report of the American College of Cardiology/American Heart Association Task Force on Clinical Practice Guidelines J Am Soc Hypertens. 2018; 12: 579.e1-579.e73. doi: 10.1016/j.jash.2018.06.010.

[S4] Pickering TG, Hall JE, Appel LJ, et al. Recommendations for blood pressure measurement in humans: an AHA scientific statement from the Council on High Blood Pressure Research Professional and Public Education Subcommittee. J Clin Hypertens (Greenwich) . 2005; 7: 102-9. doi: 10.1111/j.1524-6175.2005.04377.x.
